# Improved Power System Stability Using Backtracking Search Algorithm for Coordination Design of PSS and TCSC Damping Controller

**DOI:** 10.1371/journal.pone.0146277

**Published:** 2016-01-08

**Authors:** Naz Niamul Islam, M. A. Hannan, Azah Mohamed, Hussain Shareef

**Affiliations:** 1 Department of Electrical, Electronic and Systems Engineering, Faculty of Engineering and Built Environment, Universiti Kebangsaan Malaysia, 43600 Bnagi, Selangor, Malaysia; 2 Electrical Engineering Department, College of Engineering, United Arab Emirates University, F-1 Building, Level 1, P.O. Box 15551, Al Ain, United Arab Emirates; University of California Berkeley, UNITED STATES

## Abstract

Power system oscillation is a serious threat to the stability of multimachine power systems. The coordinated control of power system stabilizers (PSS) and thyristor-controlled series compensation (TCSC) damping controllers is a commonly used technique to provide the required damping over different modes of growing oscillations. However, their coordinated design is a complex multimodal optimization problem that is very hard to solve using traditional tuning techniques. In addition, several limitations of traditionally used techniques prevent the optimum design of coordinated controllers. In this paper, an alternate technique for robust damping over oscillation is presented using backtracking search algorithm (BSA). A 5-area 16-machine benchmark power system is considered to evaluate the design efficiency. The complete design process is conducted in a linear time-invariant (LTI) model of a power system. It includes the design formulation into a multi-objective function from the system eigenvalues. Later on, nonlinear time-domain simulations are used to compare the damping performances for different local and inter-area modes of power system oscillations. The performance of the BSA technique is compared against that of the popular particle swarm optimization (PSO) for coordinated design efficiency. Damping performances using different design techniques are compared in term of settling time and overshoot of oscillations. The results obtained verify that the BSA-based design improves the system stability significantly. The stability of the multimachine power system is improved by up to 74.47% and 79.93% for an inter-area mode and a local mode of oscillation, respectively. Thus, the proposed technique for coordinated design has great potential to improve power system stability and to maintain its secure operation.

## Introduction

Power system oscillation is one of the fundamental reasons behind unexpected power blackout incidents. Any system upset caused by a fault or series of faults may lead to the origination of oscillations in a power system [1]. In an interconnected power system, such oscillations occur in the frequency range of 0.1–3.0 Hz [2]. These oscillations are usually classified as local modes and inter-area modes [1–2]. Generators of nearby regions or the same region oscillate against each other because of local modes of oscillation. In contrast, the inter-area modes are the oscillations of coherent generators of different regions connected through very long tie lines [1–2]. In general, inter-area modes of oscillation are quite difficult to observe compared to local modes of oscillation because they are often undetected by the system operators [2–3]. Therefore, these modes are more dangerous and convey the oscillations from one region to another through weak and long tie lines of power systems [4]. To provide effective damping over both local and inter-area modes of oscillation, the coordination of the power system stabilizer (PSS) and thyristor-controlled series compensation (TCSC) controllers is recommended [5]. However, an improperly coordinated design of these controllers may cause instability in the power system [6]. Thus, the coordination of the PSS and TCSC controllers is a design problem for the safe operation of the modern multimachine power system.

Over the last few years, many optimization techniques have been applied to conduct the coordinated design of PSS and TCSC power oscillation damping (POD) controllers [7–9]. The efforts were mainly for the application of deterministic techniques and heuristic techniques to improve the damping performance of the overall power system. Deterministic techniques, such as sequential quadratic programming (SQP), have been applied for coordination design using a single-objective function formulation [7]. However, this type of optimization technique is very sensitive to the selection of the initial assumed point, and thus, the optimum solution is not achieved easily. To overcome the coordinated design complexity of damping controllers, heuristic algorithms have been used extensively over the last few years [8–11]. Heuristic algorithms, such as simulated annealing (SA), were introduced for coordination design in single-machine infinite-bus (SMIB) power systems [10]. The presented research also considered a single-objective function for optimizing the parameters of coordinated damping controllers. Later on, a multimachine power system, such as a 2-area 4-machine system, was considered for the coordinated control of damping controllers [8]. In that literature, particle swarm optimization (PSO) was proposed to improve the performance of the controller parameter optimization. Other research has also been conducted using PSO in SMIB power systems [11]. However, PSO has various control parameters, and their improper selection may significantly affect the optimal solution of coordinated damping control. As an alternative option, the bacteria foraging optimization algorithm (BFOA) has also been demonstrated for the efficient design of coordinated controllers in a 3-machine 9-bus power system model [9]. However, the presented approach avoided many parameters to simplify the optimization complexity. In addition, BFOA shows poor convergence for larger constrained problems. To address this problem, an improved version of BFOA was presented in another research study [12]. Recently, a hybrid optimization algorithm that combines modified PSO and a genetic algorithm (GA) has been applied to enhance power system stability using the coordination of the PSS and TCSC controllers [13]. However, hybrid algorithms are difficult to implement and may show other contingencies, causing adverse optimization solutions.

The application of various optimization techniques has already been proposed for the coordinated design of PSS and TCSC controllers. The presented approaches were conducted using single-objective functions in small-sized test power systems. However, the design of damping controllers using multi-objective functions is more efficient than that using single-objective functions [14]. On the other hand, the number of optimization parameters in a small-sized test power system is relatively few. In addition, the consideration of a reduced number of optimizing parameters may not provide robust damping by the coordinated control schemes. Thus, the previously presented researchers have not covered the robust performance of coordinated damping control in the case of a large power system. However, modern power systems are very large, and the nature of power system oscillations has become very complicated [2]. In general, the optimizing parameters are huge for large power systems. In such cases, traditionally used optimization techniques may not succeed in finding the optimum solution of coordinated damping controls because of their aforementioned common pitfalls. Therefore, the safety of modern power systems requires looking for new and efficient optimization algorithms. The backtracking search algorithm (BSA) is one of the metaheuristic algorithms recently developed [15]. The performance of BSA in handling optimization with a large number of parameters has been proven to be efficient [15]. Therefore, this research investigates the feasibility of BSA application for the robust coordinated design of PSS and TCSC controllers in a large multimachine power system.

## Multimachine Power System with Damping Controllers

**Fig 1:**
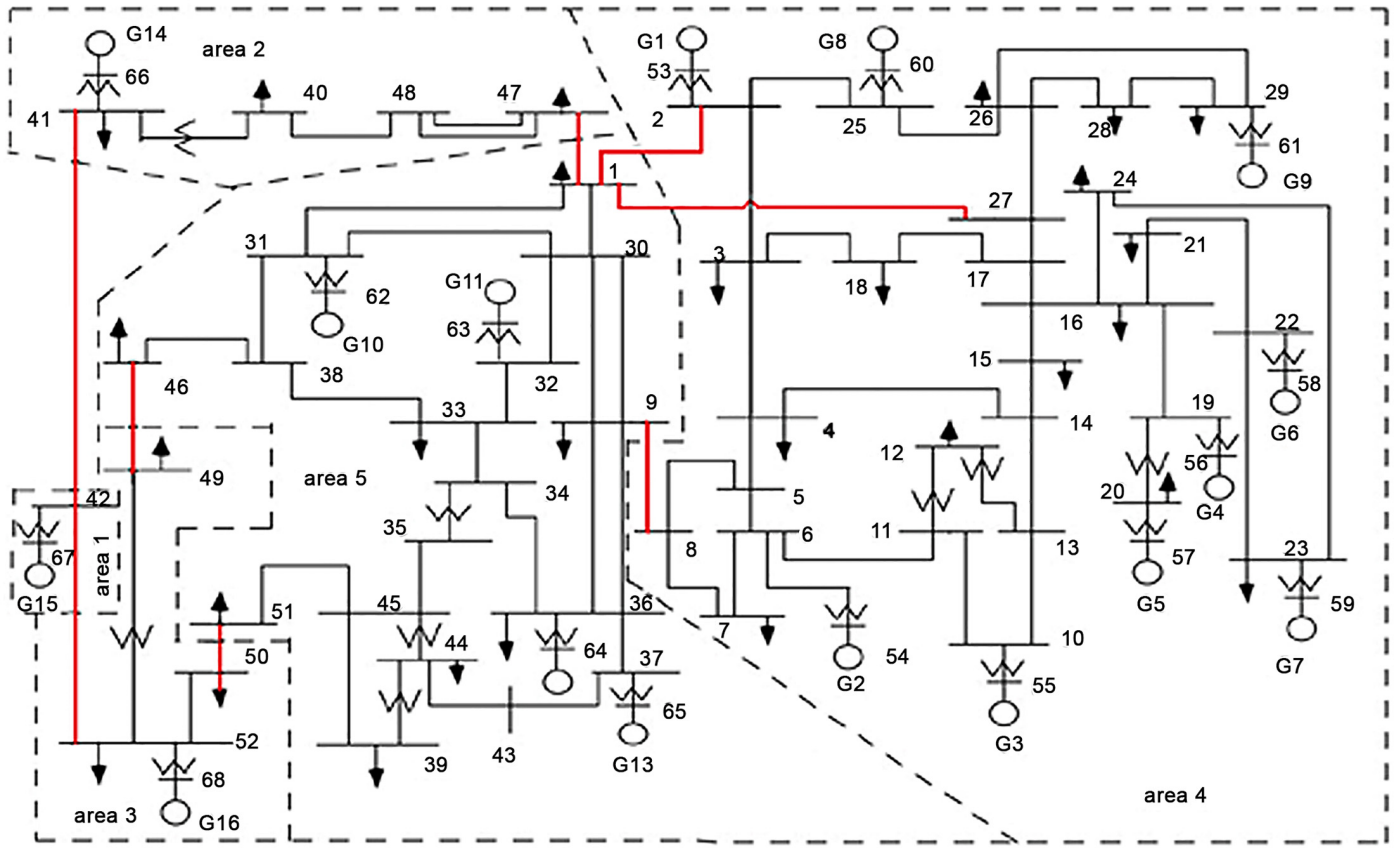
Single line diagram of the 5-area 16-machine power system.

**Fig 2:**
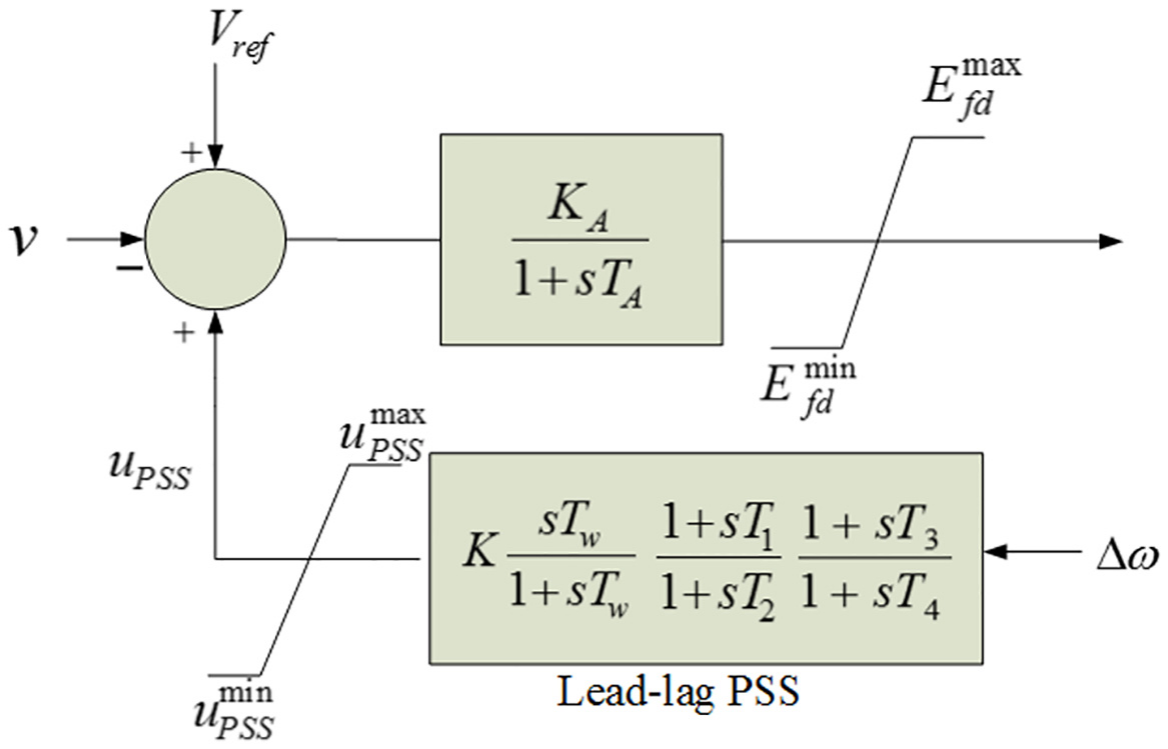
Power system stabilizer with the IEEE type-ST1 excitation system.

This study is intended to investigate the design of coordinated controllers in a relatively large power system. To do that, a benchmark 5-area 16-machine power system has been taken, in which the optimization of controllers is coordinated in such a way as to maximize the overall system damping over power system oscillations. The single line diagram of the 5-area power system is shown in Fig 1. In this system, there are 5 regions and 68 buses according to modal analysis [2]. Generator 15 is in area 1, Generator 14 is in area 2, Generator 16 is in area 3, Generators 1–9 are in area 4 and Generators 10–13 are in area 5. All 5 areas are connected to each other through a total of 8 tie lines as marked by the color red in Fig 1. The design of damping controller is associated with the study of power system stability. Therefore, it is conducted in the linear (linear time-invariant state-space) and non-linear models of a power system [2, 7–9]. In order to conduct the optimization of damping controllers, the linearized model of the power system is used to formulate the required objective function (or cost function) [7–9]. After that, the optimized parameters of damping controllers are taken to verify the design efficiency in non-linear model with the help of time-domain analysis. The complete modeling of a multimachine power system in linear and non-linear forms is associated with proper and accurate mathematical representation which is very tedious and complicated work [16]. Therefore, in this research, the power system toolbox (PST) (version 3) has been adopted to simplify the overall modeling burden, and the focus is concentrated mainly on the coordination design of damping controllers. This toolbox can be obtained along with Roger’s book [2]. The toolbox can also be downloaded via a website link [17]. The PST contains the linear model in the form of linear time-invariant (LTI) state space approach and non-linear model of multimachine power system. This toolbox was developed following IEEE standard models of power system components [18]. Therefore, it is considered as one of the standard and popular toolbox commonly used for the study of power system oscillations [19–20]. In this research, required models of damping controllers were implemented in PST according to the instruction given in PST manual [21]. In addition, the essential models of damping controllers are summarized and explained for fundamental understanding. The excitation system of each synchronous generator is modeled in the IEEE type-ST1 equipped with a PSS, as shown in Fig 2.

**Fig 3:**
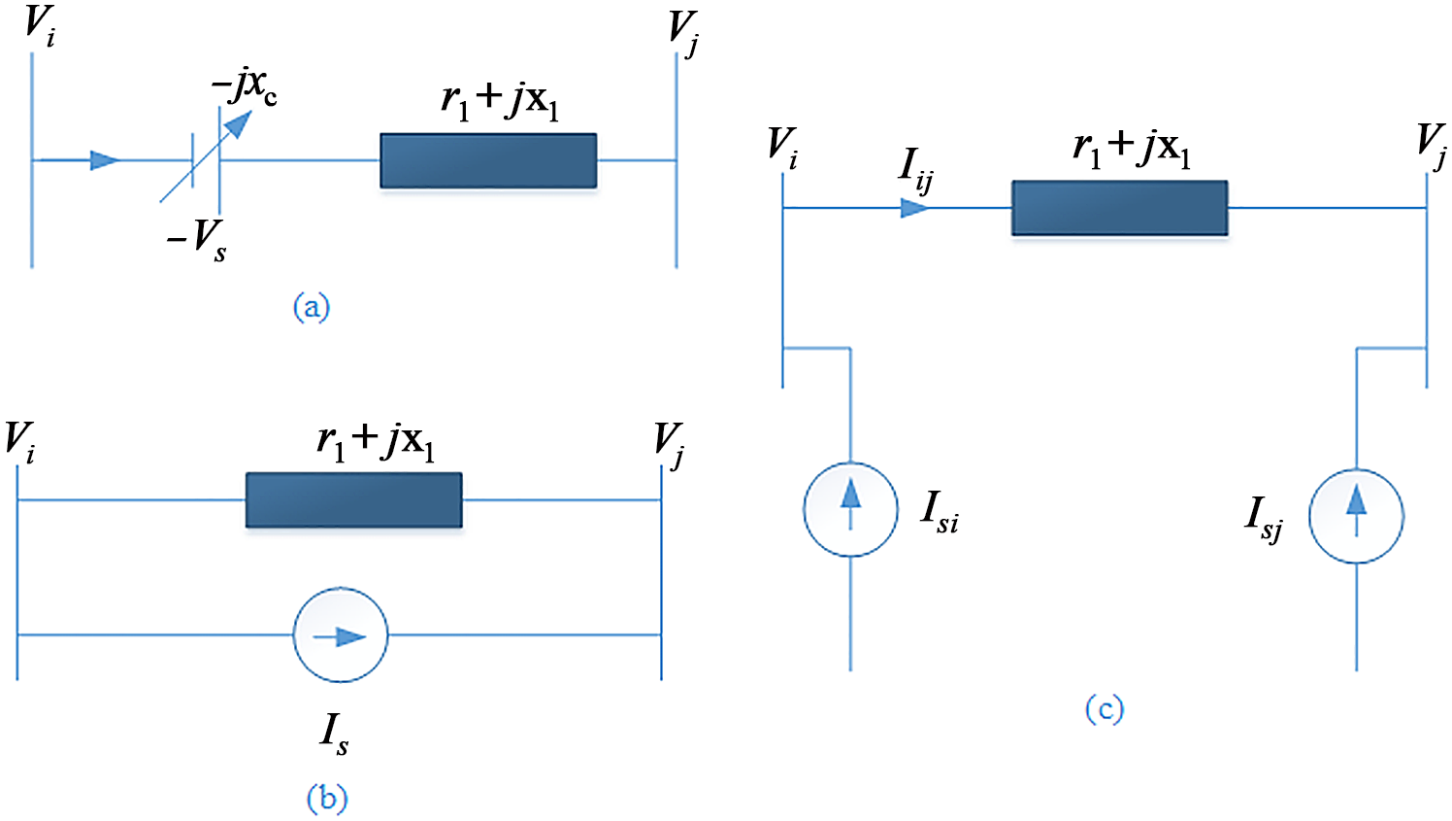
Modeling of a TCSC: a) TCSC location in a transmission line. b) Representation of the voltage source in the current source. C) Current injection model.

In the 5-area system, a TCSC is placed between Buses 50 and 51 [7]. It is modeled in the current injection model, and the required model representation is shown in Fig 3 [8]. The model of the TCSC is represented in the form of a variable reactance between two Buses, *i* and *j*. The dynamic model equations of a TCSC in a transmission line are presented using Eqs 1–4.

$$I_{se}=\frac{V_{i}-V_{j}}{r_{l}+j(x_{l}-x_{c})}$$ (1)

$$V_{s}=-jX_{c}I_{se}$$ (2)

$$I_{s}=\frac{V_{s}}{r_{l}+jx_{l}}=-\frac{jx_{c}I_{se}}{r_{l}+jx_{l}}$$ (3)

$$I_{si}=\frac{jx_{c}}{r_{l}+jx_{l}}\cdot \frac{V_{i}-V_{j}}{r_{l}+j(x_{l}-x_{c})}=-I_{sj}$$ (4)

**Fig 4:**
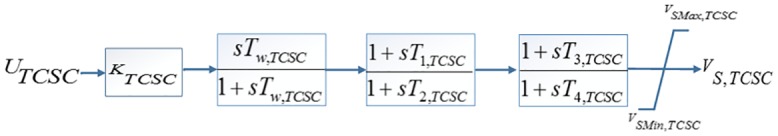
Block diagram of the TCSC POD controller.

The POD structure is taken as the damping controller of the TCSC shown in Fig 4. It is identical to the structure of a PSS. The purpose of the controllers is to provide required phase compensation to the input of the TCSC in a way that provides sufficient damping over oscillations of the power system. The required phase compensation is ensured by limiting over- and under-compensation through the use of a limiter, as shown in Fig 4.

For both controllers (PSS and TCSC POD), two stages of lead-lag compensator blocks are usually considered to manage the required phase compensation. From Figs 2 and 4, the proper selection of gain (*K*) and time constants (*T*_*1*_, *T*_*2*_, *T*_*3*_, *T*_*4*_) are the design problem for the coordination of the PSS and TCSC controllers. The design of the controllers is a constrained optimization problem that is subjected to the constraints shown in Eq 5 for the PSS controller and in Eq 6 for the TCSC controller.

$$\begin{array}{l} K_{\text{min},PSS}\leq K_{PSS}\leq K_{\text{max},PSS}\\ T_{1,\text{min},PSS}\leq T_{1,PSS}\leq T_{1,\text{max},PSS}\\ T_{2,\text{min},PSS}\leq T_{2,PSS}\leq T_{2,\text{max},PSS}\\ T_{3,\text{min},PSS}\leq T_{3,PSS}\leq T_{3,\text{max},PSS}\\ T_{4,\text{min},PSS}\leq T_{4,PSS}\leq T_{4,\text{max},PSS} \end{array}$$ (5)

$$\begin{array}{l} K_{\text{min},TCSC}\leq K_{TCSC}\leq K_{\text{max},TCSC}\\ T_{1,\text{min},TCSC}\leq T_{1,TCSC}\leq T_{1,\text{max},TCSC}\\ T_{2,\text{min},TCSC}\leq T_{2,TCSC}\leq T_{2,\text{max},TCSC}\\ T_{3,\text{min},TCSC}\leq T_{3,TCSC}\leq T_{3,\text{max},TCSC}\\ T_{4,\text{min},TCSC}\leq T_{4,TCSC}\leq T_{4,\text{max},TCSC} \end{array}$$ (6)

## Problem Formulation

**Fig 5:**
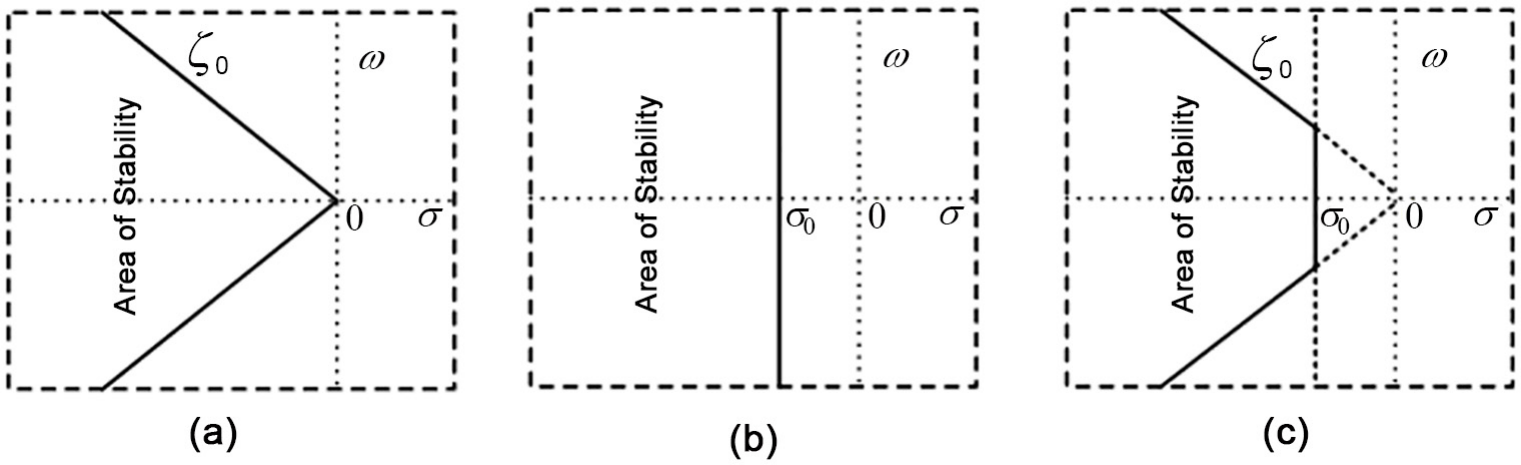
Formulation of the multi-objective D-shaped cost function for stability analysis in the s-plane. a) Stability defined by the expected damping ratio; b) Stability defined by the expected damping factor; c) Combination of the expected damping ratio and factor.

A power system model consists of a set of ordinary differential equations (ODE) for the dynamic model and a set of algebraic equations for the system network model. To determine the system stability, the final model of the entire power system is linearized around an operating point, as shown in Eq 7. The linearization of the power system is conducted via a linear time-invariant (LTI) state space method [8–9].

$$\begin{array}{l} \dot{x}=Ax+Bu\\ y=Cx+Du \end{array}$$ (7)

where *A* is the system state matrix, *B* is the input matrix, *C* is the output matrix and *D* is the feed-forward matrix. Then, the system eigenvalues are determined from the state matrix (*A*). The corresponding damping factor and damping ratio are also calculated using Eqs 9 and 10, respectively. For enhanced damping performance, the linearized model-based D-shaped objective function has been proven to be efficient [14]. Therefore, the problem formulation of the coordinated design of the PSS and TCSC controllers is introduced in the form of the D-shaped stability regions shown in Fig 5. The mathematical formulation of the objective function is expressed in Eq 11.

$$\text{System eigenvalues},\;\lambda_{i}=eig(A)$$ (8)

$$\text{Damping factors},\;\sigma_{i}=real(\lambda_{i})$$ (9)

$$\text{Damping ratios},\;\zeta_{i}=-\frac{\sigma_{i}}{\sqrt{\sigma_{i}^{2}+\omega_{i}^{2}}}$$ (10)

$$\text{Objective function},\;f=\sum_{j=1}^{np}\sum_{\sigma_{ij}\geq \sigma_{0}}{\left(\sigma_{0}-\sigma_{ij}\right)}^{2}+a\sum_{j=1}^{np}\sum_{\zeta_{ij}\leq \zeta_{0}}{\left(\zeta_{0}-\zeta_{ij}\right)}^{2}$$ (11)

In the coordinated design, the power system stability is ensured by minimizing the objective function (*f*) subjected to the design constraints in Eqs 5 and 6, in which the unstable and lightly damped electromechanical modes are forced to relocate into the region of stability in the complex s-plane.

## Backtracking Search Algorithm

BSA is an efficient metaheuristic algorithm for multimodal optimization problems that was developed by Civicioglu in 2012 [15]. The simplified and unique structure of BSA has encouraged the solving of real-world complex optimization problems. In BSA, there is only one control parameter, and the optimization solution is not sensible for its unaware selection. In addition, BSA has the capability to handle a large number of optimization parameters without compromising the solution quality. Unlike other metaheuristic algorithms, the solution obtained using BSA is very much consistent, which is very important for power system applications. It is a population-based algorithm. It uses a large number of the population to move toward an optimum solution. The unique concepts of this algorithm are the historical population and map matrix. The path for the optimum solution is defined by the historical population for each movement. To overcome the local minima traps, BSA uses the historical population to explore a new solution field as well as exploit better solutions within a solution field. The map matrix is used to refine the solution in an exploitation search. Therefore, the optimum solution is guaranteed based on simultaneous search exploration and exploitation. The working principle of BSA is composed of five main steps, as described below [15]:

### Step 1: Initialization

The primary population and historical population of the BSA are generated based on a uniform distribution (∪) within the boundary constraints. If the optimization problem dimension and population size are *D* and *N*, respectively, for the optimization, then each individual of the main population (*Pop*_*i*_) and historical population (*His*_*i*_) are initialized as follows:

$$\text{Primary population},\;Pop_{n,d}\sim \cup (low_{d},up_{d})$$ (12)

$$\text{Historical population},\;His_{n,d}\sim \cup (low_{d},up_{d})$$ (13)

$$\text{Fitness value},\;y_{pop}\;=\;f(Pop)$$ (14)

where *n*∈{1,2,3,…,*N*} and *d*∈{1,2,3,…,*D*}. The search space for controller parameters are defined by the two row vectors *low*_*d*_ and *up*_*d*_.

### Step 2: Selection-I

The historical population is updated based on Eqs 15 and 16.

$$\begin{array}{cccc} if & a<b & then & His:=Pop|a,b\sim \cup (0,1) \end{array}$$ (15)

His:=permuting(His) (16)

### Step 3: Mutation

The initial value of the trial population is known as the mutant.

Initial trial population, Mut=Pop+R.(His−Pop) (17)

Standard Brownian-walk, R=3⋅randn (18)

where *randn* is the build-in MATLAB^®^ function for generating normally distributed values (0~1).

### Step 4: Crossover

This part generates the final form of the trial population that complies with the optimization boundary constraints. The crossover of BSA consists of two parts:

#### Step 4 (a): Part-1

This process controls the number of elements of individuals to be mutated by generating a binary *map* matrix having the same size as *Pop*. It decides which individuals will be manipulated and which will be unchanged in the mutation process. The formulation of the *map* matrix is based on Eqs 19 and 20.

$$\text{Map matrix initialization},\;map_{\left(1:N,1:D\right)}=1$$ (19)

$$\begin{array}{cc} \text{Map matrix formulation},\; & \begin{array}{l} \text{if}\begin{array}{l} a\langle b|a,b\sim U(0,1)\text{then} \end{array}\\ \text{ for }n\text{ from }1\text{ to }N\text{ do}\\ \;map_{n,u_{\left(1:[mixrate.rand.D]\right)}}=0|u=permuting(\langle 1,2,3,\ldots ,D\rangle )\\ \text{ end}\\ \text{else}\\ \begin{array}{cc} & \text{for }\begin{array}{l} n\begin{array}{l} \text{ from}\begin{array}{l} 1\begin{array}{l} \text{to}\begin{array}{l} N\begin{array}{l} \text{do, }\begin{array}{l} map_{n,randi(D)}=0,\begin{array}{l} \text{ end} \end{array} \end{array} \end{array} \end{array} \end{array} \end{array} \end{array} \end{array} \end{array}\\ \text{end} \end{array} \end{array}$$ (20)

$$\text{Final trial population},\;Trial_{n,d}:=\{\begin{array}{ll} Pop_{n,d} & if\;map_{n,d}=1\\ Mut_{n,d} & if\;map_{n,d}=0 \end{array}$$ (21)

#### Step 4 (b): Part-2

The boundary condition of the trial population is checked and updated using Eq 22

$$\;Trial_{n,d}=low_{d}+rand\cdot (up_{d}-low_{d}),\text{ if }\left(Trial_{n,d}<low_{d}\right)\text{ or }\left(Trial_{n,d}>up_{d}\right)$$ (22)

### Step 5: Selection-II

In this step, the trial population from Eq 22 is used to evaluate the cost function again, and the corresponding fitness values are determined using Eq 23. The calculated fitness values are then compared with the fitness values (*y*_*pop*_) of the main population (*Pop*) to query any fruitful discovery, as shown in Eqs 23 and 24.

$$\text{Trail population fitness values}y_{trial}=f(Trial)$$ (23)

$$Pop_{n}=Trial_{n},if\begin{array}{c} y_{n,Trial}^{}>y_{n,Pop} \end{array}$$ (24)

For further BSA details, interested readers are referred to its original paper [15]. The following section will depict the major steps to explain the application of this algorithm to coordination damping improvement.

## BSA Implementation for Coordination of PSS and TCSC Controllers

**Fig 6:**
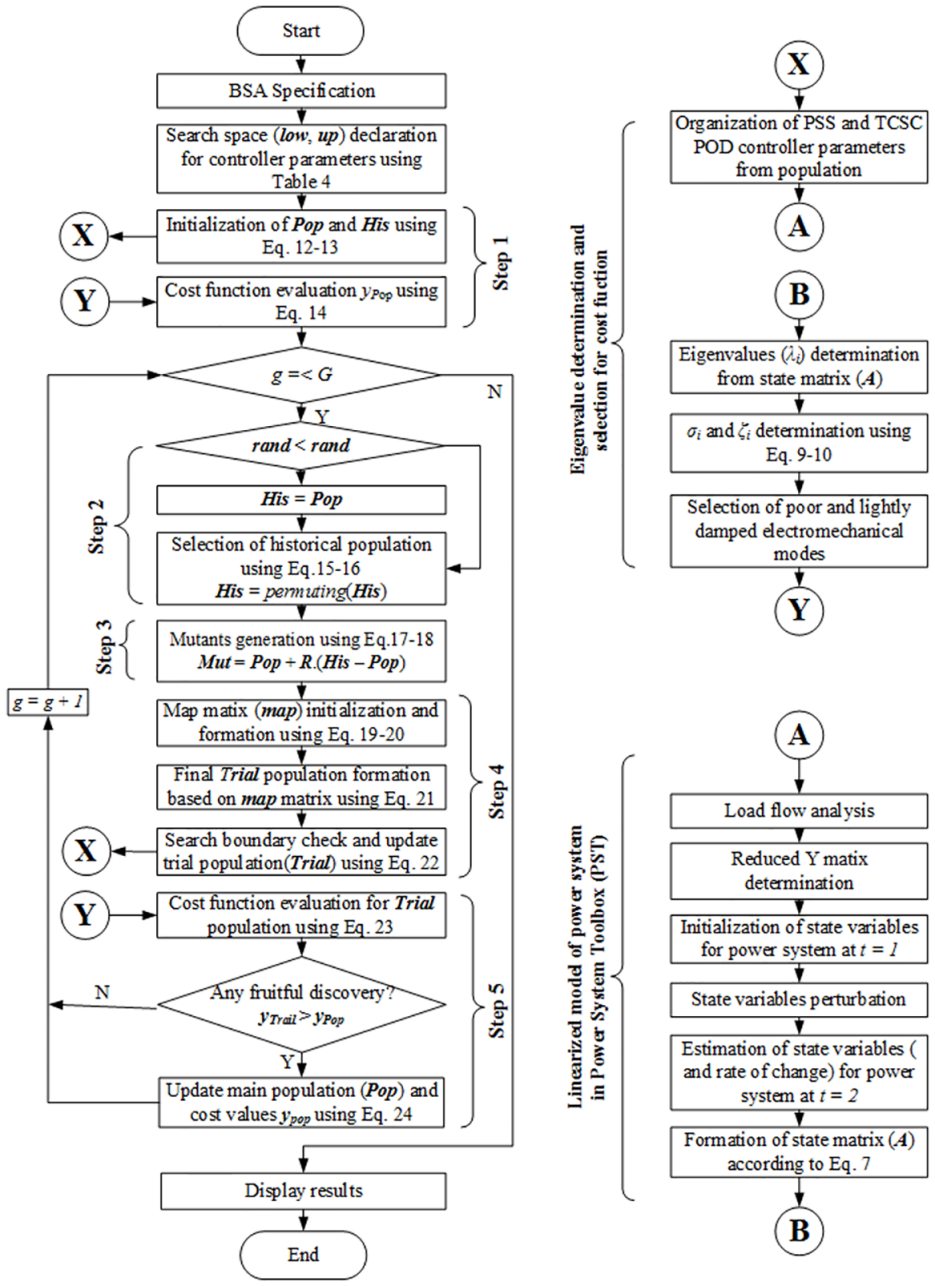
BSA implementation block diagram for the coordination design of the PSS and TCSC controllers.

The complete coordinated design of the PSS and TCSC POD controllers using the BSA technique is categorized into three major sections. The use of the power system toolbox simplifies the overall process. For simplicity and understanding, the overall design procedures are depicted in Fig 6. The major work functionalities are summarized as follows:

### Section 1

This section is associated with the core steps of the BSA technique.

- Part 1: First, the BSA is specified by its population size, dimension size, number of generations, control parameter, etc.
- Part 2: The boundary limits for the search space are defined here using two row vectors (*low*, *up*). These two vectors correspond to the controller parameter limits.
- Part 3: In this part, Step 1 from BSA (as shown in Fig 6) is performed. The following sections, 2–3, are also incorporated along with this part.
- Part 4: Steps 2–5 from BSA are followed in this part.
- Part 5: Optimized results are obtained when the number of executions meets the maximum generation.

### Section 2

This section addresses the determination and selection of eigenvalues from the state matrix (*A*). The purpose of this section is to sort out the data for calculating the cost function value using the given population.

- Part 1: The input (i.e., the PSS and TCSC controller parameters) of this section is the BSA population. This part is associated with the organization of the controller parameters according to the data format of the power system toolbox.
- Part 2: The organized controller’s data, along with the considered power system data, are processed to obtain the system state matrix (*A*) of the linear power system using the power system toolbox.
- Part 3: The eigenvalues (*λ*_*i*_) are determined from the system state matrix (*A*).
- Part 4: The damping factor (*σ*_*i*_) and damping ratio (*ζ*_*i*_) are calculated for each eigenvalue using Eqs 9 and 10.
- Part 5: The eigenvalues having a negative damping factor (*σ*<0) and a very high damping ratio (*ζ*>0.95) are considered to be zero eigenvalues, and those have no influence on system oscillation. Non-zero eigenvalues located outside of the D-shaped stability region are considered to calculate the cost function value using Eq 11.

### Section 3

This section addresses the toolbox used for the power system linearized model.

- Part 1: Import the data file for the test power system
- Part 2: Run the load flow analysis
- Part 3: Determine the reduced admittance (Y) matrix
- Part 4: Initialize the state variables (*state*_*i*_), their rates of change (*dstate*_*i*_) and the system state matrix (*A*) for time step *t =* 1.
- Part 5: At time step *t* = 2, the state variables are sequentially perturbed and the value of the corresponding *dstate*_*i*_ is estimated. The system state matrix (*A*) is recalculated for each perturbation of the system state variables. A perturbation matrix contains the sequence index of the system state variables through which the perturbations of the state variables are executed one after another. The perturbation matrix is used in the form of a sparse matrix. As the formulation of the state matrix is based on the perturbation of every state variable, the consideration of the system operating conditions is no longer required during the optimization design of the damping controllers in the linearized model of the power system. Therefore, the value of *np* (operating conditions) in Eq 11 is set to 1.
- Part 6: The formation of the final state matrix (*A*) is performed in this part.

## Results and Discussion

In the study, simulations were conducted in two models, a linearized model and a non-linear model of the power system. The required data for the 5-area power system were taken from the power system toolbox (version 3) [2]. The optimization of the coordinated controllers (PSS and TCSC POD) has been conducted in the linearized model using the proposed BSA technique. The number of optimizing parameters for each PSS and TCSC controller are 5. Hence, a total of 85 (5x16+5) parameters were optimized in the 5-area system by minimizing the formulated D-shaped objective function. The time constant for the washout block, *T*_w_, was set to 10 s for both the PSS and TCSC POD [9, 14]. Other design parameters considered in the optimization are presented with the considered values in the Appendix. The design constraints of the optimizing parameters are shown in Table 1. Detailed analysis is conducted here to assess the damping performance of the designed coordinated controls. To check the design robustness of the coordinated damping, a severe 3-phase fault is ap2plied to destabilize the power system during the simulation in the non-linear model. Then, the damping performance was recorded in terms of the settling time. For a comparative and comprehensive analysis, PSO has also been selected along with the BSA technique to investigate the improvement of the design performance.

**Table 1:** Optimization parameter constraints.

Limits	PSS					TCSC POD Controller				
	*K*	*T*_*1*_	*T*_*2*_	*T*_*3*_	*T*_*4*_	*K*	*T*_*1*_	*T*_*2*_	*T*_*3*_	*T*_*4*_
*Low*	0.01	0.01	0.01	0.01	0.01	0.01	0.01	0.01	0.01	0.01
*Up*	50	2	2	2	2	50	2	2	2	2

### Statistical Analysis

**Fig 7:**
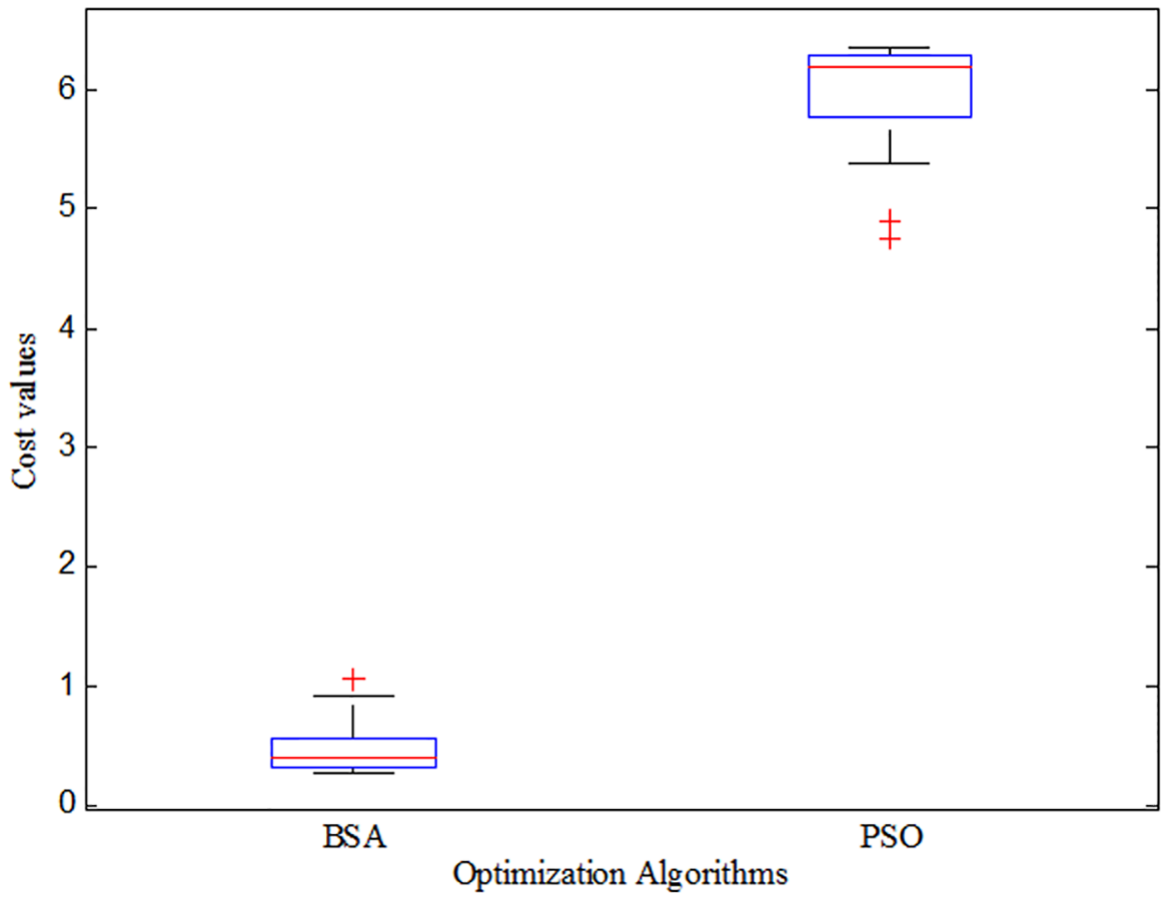
Statistical box and whisker plots of each algorithm obtained from 15 individual simulation in linear model of multimachine power system.

The robustness of an optimization algorithm are usually justified based on its solution quality and solution consistency. The best values obtained using an algorithm represents the solution quality, while the solution consistency is also important to show the reliability of an algorithm’s ability to find a good solution within limited optimization runs. Therefore, appropriate and rigorous statistical analyses are preferred to verify the efficiency of BSA technique in terms of solution quality and consistency for the improvement of power system stability. In this research, the statistical box and whisker plot is used as a suitable tool to analyze the solution quality and consistency of the proposed BSA technique. To conduct detailed statistical analysis, optimization simulations were conducted 15 times for each algorithm in the linearized model of the power system. The fitness values of 15 independent optimization runs were used to draw box and whisker plots with the help of MATLAB^®^ built-in function. The box and whisker plots of the BSA and PSO are shown in Fig 7. According to the box and whisker plots shown in Fig 7, the solution quality of BSA-based design is much better for all quartiles than those of the PSO-based design. In this case, the proposed BSA technique outperforms PSO technique for finding quality solutions. In addition, the solution obtained by BSA technique is much consistent spread in the narrower region (0.3070–0.5599) compared with PSO (5.7821–6.2898) as shown in Fig 7. Therefore, the BSA technique has the ability to find a consistent solution than PSO for the application of large power system optimization.

The fitness values from the simulations are summarized in terms of best, mean, median worst, and standard deviation in Table 2. It is observed that the fitness values using the BSA technique are significantly lower than those using the PSO technique. The best solutions obtained using BSA and PSO have fitness values of 0.2744 and 4.7645, respectively. In addition to these, the mean, median, worst and standard deviation of the solutions using BSA are much less than those using PSO. Therefore, this indicates that the BSA technique is far superior and yields better solutions compared to the PSO technique.

**Table 2:** Best solutions (cost values) obtained by each algorithm from 15 individual runs.

System	Objective function values	
	BSA	PSO
Best	0.2744	4.7645
Mean	0.4816	5.9176
Median	0.3946	6.1833
Worst	1.0642	6.3481
Standard deviation	0.2321	0.5166

### Time Domain Analysis

According to the fitness values obtained, the best optimized parameters of the coordinated damping controls were selected to investigate the design performance. The controller parameters associated with the best fitness value are listed in Table 3. The design performances were analyzed via time-domain analysis in the non-linear model simulation of the power system. Damping performance is investigated in local modes as well as inter-area modes of power system oscillations. The local modes W10-W11 and W13-W10, and the inter-area modes W13-W16 and W10-15 are considered in this study. For each oscillation mode, the performance of the BSA-based design is compared with that of the PSO-based design.

**Table 3:** Best optimized parameters using each optimization algorithm for the 5-area system.

Algorithm	PSS						TCSC POD Controller				
	Number	*K*	*T*_*1*_	*T*_*2*_	*T*_*3*_	*T*_*4*_	*K*	*T*_*1*_	*T*_*2*_	*T*_*3*_	*T*_*4*_
*BSA*	*PSS1*	9.7613	0.4525	0.7466	0.9097	0.9759	18.7846	1.6271	1.0494	0.9543	0.0454
*BSA*	*PSS2*	3.4994	0.8642	0.0312	0.8802	0.7184					
*BSA*	*PSS3*	12.0236	0.1096	1.0138	1.0514	0.0293					
*BSA*	*PSS4*	7.6442	0.5322	0.8305	0.9017	1.1790					
*BSA*	*PSS5*	0.9369	1.1772	0.1076	0.8304	0.6266					
*BSA*	*PSS6*	0.4503	0.8450	0.9997	0.9678	0.6574					
*BSA*	*PSS7*	6.4102	0.3739	0.9504	0.2925	0.0101					
*BSA*	*PSS8*	1.7368	0.5965	0.0502	0.6030	0.7723					
*BSA*	*PSS9*	8.9462	0.3028	0.0563	0.4471	0.9846					
*BSA*	*PSS10*	4.4567	0.7353	0.0528	0.6428	0.8428					
*BSA*	*PSS11*	2.0962	0.5073	0.0159	0.5088	0.9325					
*BSA*	*PSS12*	1.4507	1.0991	0.8038	0.8774	0.0677					
*BSA*	*PSS13*	18.5628	1.0955	0.6599	0.5693	0.3004					
*BSA*	*PSS14*	12.6816	1.0372	0.9822	0.7264	0.8620					
*BSA*	*PSS15*	8.9038	0.9058	0.9149	1.4136	0.7164					
*BSA*	*PSS16*	14.8952	0.7179	0.4319	0.9149	0.9815					
*PSO*	*PSS1*	7.4796	0.8329	0.8247	0.5677	0.0786	11.691	0.2913	0.3865	1.4313	0.6229
*PSO*	*PSS2*	4.1629	0.7910	0.1020	0.6587	1.1224					
*PSO*	*PSS3*	4.1049	1.0510	0.4939	0.5558	0.6085					
*PSO*	*PSS4*	5.1507	0.7397	0.0408	0.5298	0.7585					
*PSO*	*PSS5*	1.3900	1.2361	0.6996	0.5546	0.8911					
*PSO*	*PSS6*	1.9086	0.9429	0.7705	0.7013	0.9365					
*PSO*	*PSS7*	10.2108	0.8692	1.0375	0.8644	1.3098					
*PSO*	*PSS8*	9.3250	0.3292	0.9987	0.5302	0.0200					
*PSO*	*PSS9*	11.1997	0.4045	1.0404	0.6427	0.0335					
*PSO*	*PSS10*	6.9675	0.8119	1.6054	1.4964	0.2325					
*PSO*	*PSS11*	6.9854	0.3095	0.0124	0.4184	1.1249					
*PSO*	*PSS12*	3.3121	1.1550	0.7626	0.8992	0.0608					
*PSO*	*PSS13*	11.8973	1.0895	0.5557	0.8760	0.9437					
*PSO*	*PSS14*	14.7728	0.9819	0.6763	1.7135	0.8104					
*PSO*	*PSS15*	13.0235	0.8943	0.6299	0.4798	0.7965					
*PSO*	*PSS16*	12.8661	1.4113	0.5436	0.6029	1.4328					

**Fig 8:**
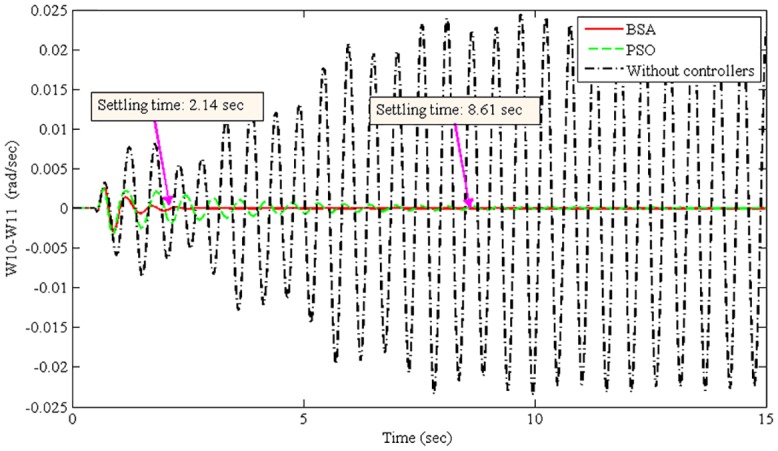
Local mode in area 5 between G10 and G11 (W10-W11) for the 3-phase fault at bus 1.

**Fig 9:**
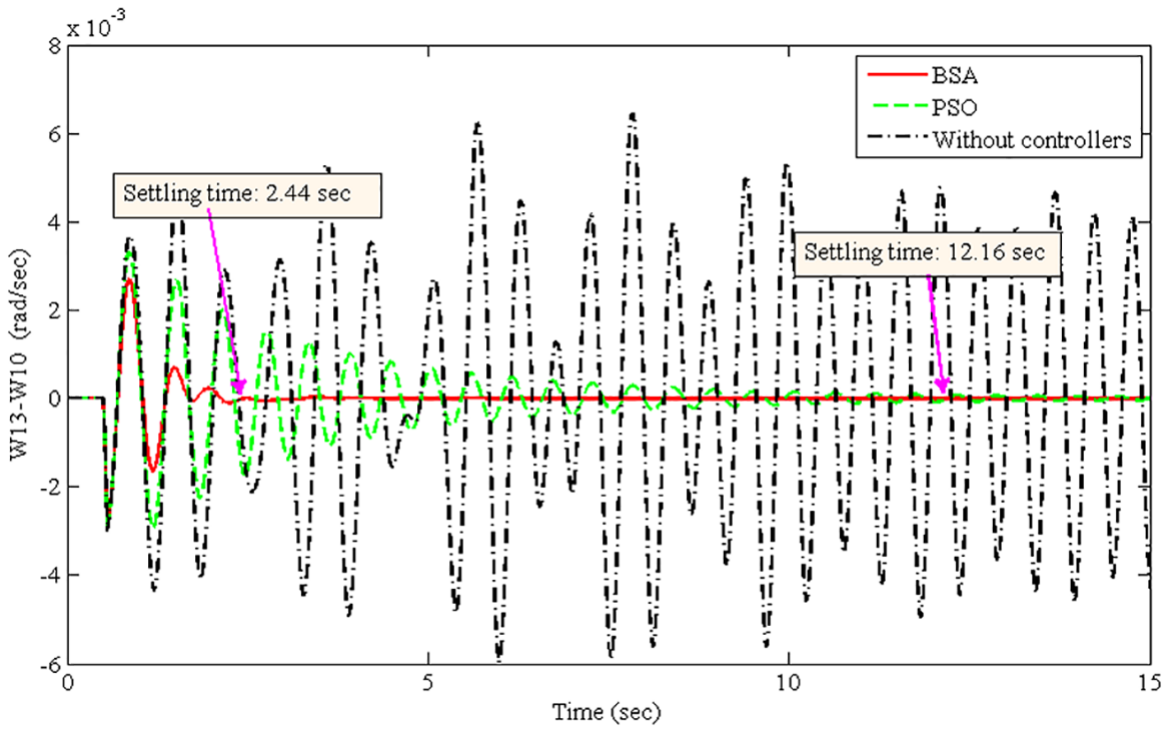
Local mode in area 5 between G13 and G10 (W13-W10) for the 3-phase fault at bus 1.

**Fig 10:**
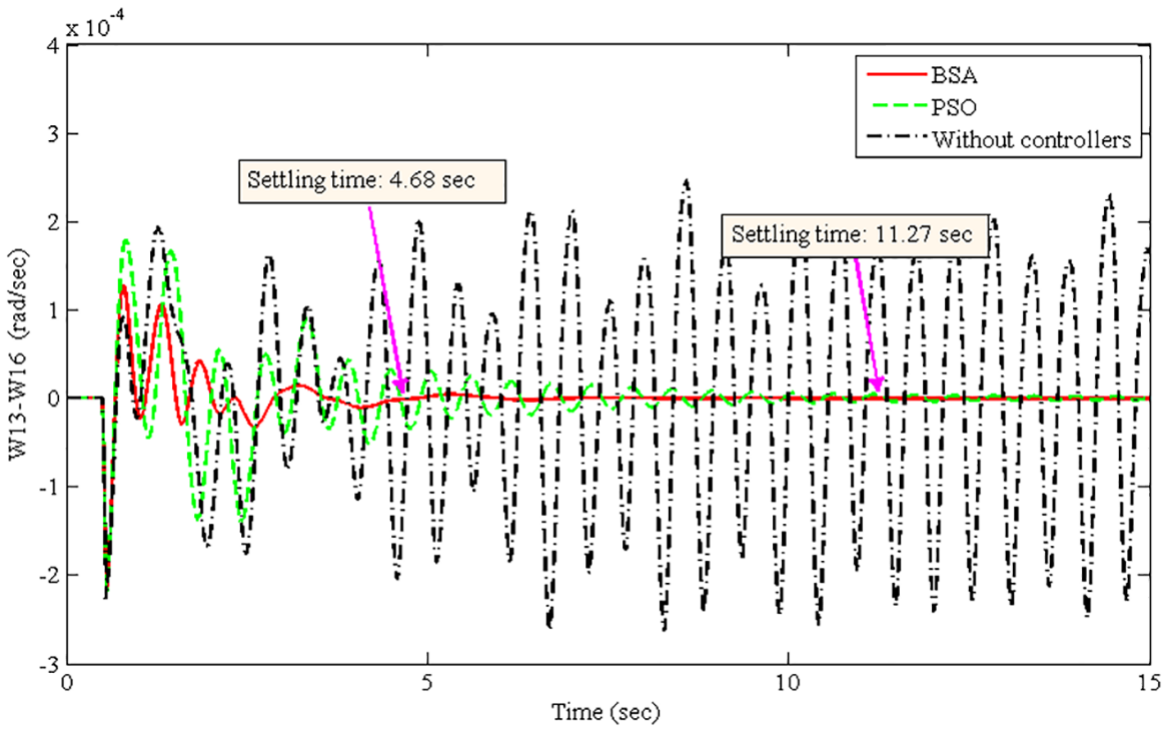
Inter-area mode between G13 in area 5 and G16 in area 3 (W13-W16) for the 3-phase fault at bus 1.

**Fig 11:**
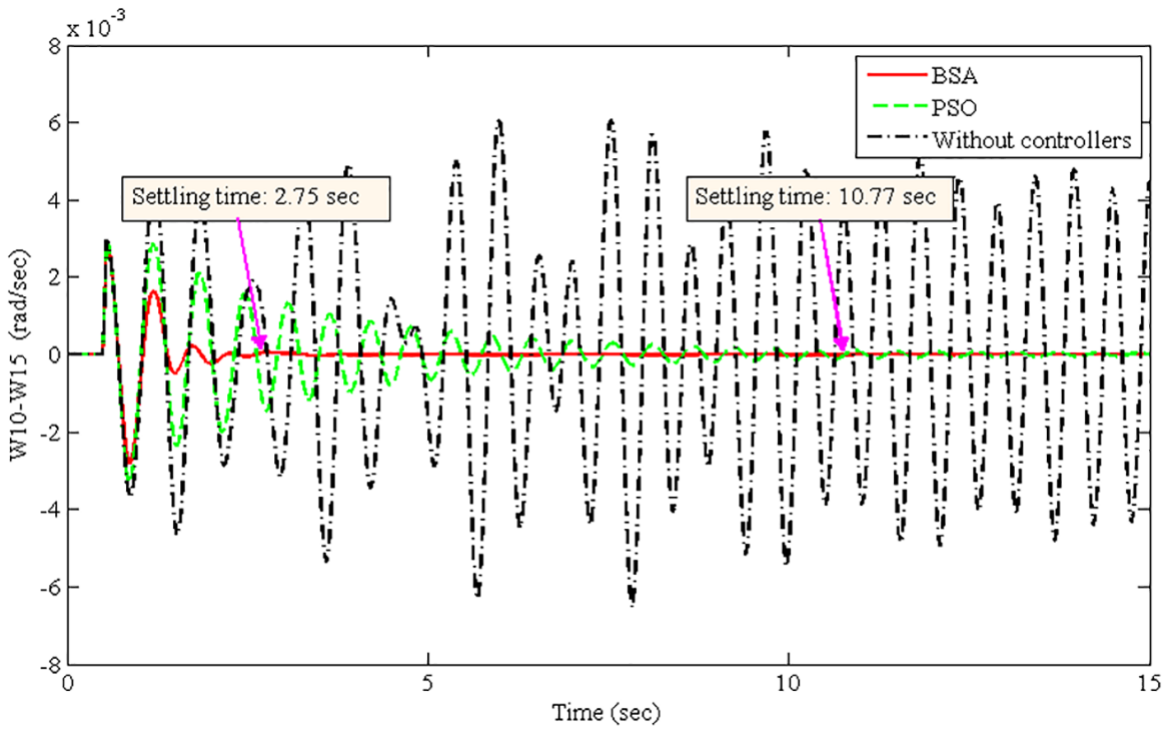
Inter-area mode between G10 in area 5 and G15 in area 1 (W10-W15) for the 3-phase fault at bus 1.

The improvement of system damping is justified in the non-linear time-domain simulations. The best optimized parameters found by the BSA- and PSO-based coordination designs are used individually to run the non-linear model of the power system. The oscillation of the power system was initiated by applying a 3-phase fault at Bus 1 (lines 1–2) after 50 ms of simulation time. The fault was cleared after 50 ms by opening lines 1–2. Power system oscillations were originated due to the severe 3-phase fault. Figs 8–11 show the damping responses obtained from the selected local and inter-area modes in the non-linear time-domain simulations. These responses were obtained by the coordinated designed controllers using the BSA and PSO techniques individually. In addition, the responses without any controllers were also recorded for the comparative damping investigation. The settling times for each oscillation mode are shown in Figs 8–11. For the BSA-based design, the damping is sufficient and quickly stabilizes the system even after being subjected to a severe 3-phase fault for all four oscillations modes. In this case, the PSO-based coordinated designed controllers show slow performance in terms of oscillation settling time. Moreover, the overshoot of oscillations is significantly reduced by the use of the BSA-based designed controller compared to the PSO-based design. For both the local and inter-area modes of oscillations, the performance of the BSA-based designed controller is superior to that of the PSO-based design.

The system stability is the primary concern of the multimachine power system. Therefore, the improvement of system stability is required to compare the proposed BSA-based design with the PSO-based design. The settling time is used here to calculate the improvement of system stability for different oscillation modes, as shown in Eq 25. Table 4 shows the summarized settling times for different modes of oscillations using the BSA- and PSO-based coordinated designs.

$$\text{Stability improvement},\;\Delta t_{s}=\frac{t_{s,PSO}-t_{s,BSA}}{t_{s,PSO}}\times 100\%$$ (25)

**Table 4:** System stability improvement in term of settling time for the BSA- and PSO-based designs.

Modes of Oscillations	Settling Time (Sec)		Stability
	BSA	PSO	Improvement (%)
Local mode, W10-W11	2.14	8.61	75.15
Local mode, W13-W10	2.44	12.16	79.93
Inter-area mode, W13-W16	4.68	11.27	58.47
Inter-area mode, W10-W15	2.75	10.77	74.47

From Table 4, the stability improvement for the local modes W10-W11 and W13-W10 are 75.15% and 79.93%, respectively. Additionally, 58.47% and 74.47% improvements have been achieved for the inter-area modes W13-W16 and W10-W15, respectively. These improvements are obtained using the BSA-based design of coordinated damping controllers over the PSO-based design. Thus, the proposed BSA-based coordinated design can enhance the stability of a multimachine power system.

## Conclusion

The coordination of PSS and TCSC POD controllers has been presented for enhanced damping performance against power system oscillations. The design problem of coordinated controllers has been formulated as a challenging optimization problem for the safety and security of the overall power system. The metaheuristic BSA technique has been applied to solve the complex formulated multi-objective function. A large multimachine benchmark power system (5-area 16-machine) has been taken into account for the design investigation of damping controllers and the system stability study. The metaheuristic PSO technique has also been considered to compare the design performances of the proposed BSA technique-based damping controllers. Based on the statistical results obtained from the linearized model optimization, the BSA technique has found more optimum solutions compared to the PSO-based technique. System stability has been studied after applying a severe 3-phase fault in non-linear time-domain simulations. The oscillation settling time and overshoot have been reduced significantly for local and inter-area modes of oscillations using the BSA-based design. In some cases, the system stability has been improved by up to 79.93% in terms of settling time. Thus, the presented BSA technique has great potential to improve the stability of interconnected modern power systems for secure and reliable operation.

## Appendix

Linear model optimization settings:

- 5-AREA System:
  - Design Setting: expected design damping factor (*σ*_0_) = -1; expected design damping ratio (*ζ*_0_) = 0.15; problem dimension (*D*) = 85;
  - Optimization Algorithm Settings:
    - BSA Settings: population size (*N*) = 25; generation (*G)* = 2000; *mixrate* = 1.0;
    - PSO Settings: population size (*N*) = 25; generation (G) = 2000; cognitive constant (*C*_*1*_) = 2; social constant (*C*_*2*_) = 2; *w*_*min*_ = 0.4; *w*_*max*_ = 0.9.
